# Genetic Targeting in Cerebellar Purkinje Cells: an Update

**DOI:** 10.1007/s12311-016-0770-4

**Published:** 2016-03-11

**Authors:** Anna Sługocka, Jan Wiaderkiewicz, Jaroslaw J. Barski

**Affiliations:** 1 Center for Experimental Medicine, School of Medicine in Katowice, Medical University of Silesia, Katowice, Poland; 2 Department of Physiology, School of Medicine in Katowice, Medical University of Silesia, Katowice, Poland; 3 Department of Pharmacology & Physiology, The George Washington University, 2300 Eye St., NW, Washington, DC 20037 USA

**Keywords:** Gene targeting, Cerebellum, Purkinje cells, Mouse

## Abstract

Since the last review paper published in Cerebellum in 2002 [1], there has been a substantial increase in the number of experiments utilizing transgenic manipulations in murine cerebellar Purkinje cells. Most of these approaches were made possible with the use of the Cre/loxP methodology and pcp2/L7 based Cre recombinase expressing transgenic mouse strains. This review aims to summarize all studies which used Purkinje cell specific transgenesis since the first use of mouse strain with Purkinje cell specific Cre expression in 2002.

## Introduction

After many years of research, there is still no consensus on the function of the cerebellum. It is accepted today that the cerebellum has a much more broad role than simply that of motor coordination. Purkinje cells (PCs), which are the sole efferent output of the cerebellum, are one of few neuronal populations that express highly specific genes.

This review will focus mainly on the Cre/loxP recombination system, which is a unique tool for Purkinje-cell specific trangenesis that employs the Purkinje cell specific gene coding L7 or pcp2 (Purkinje cells specific protein-2). This protein was described for the first time in 1988 by two independent groups and this was the starting point of Purkinje cell specific transgenesis [2, 3]. In the following text, we will use official nomenclature of pcp2 according to the Mouse Genome Informatics database at www.jax.org.

**Fig. 1:**
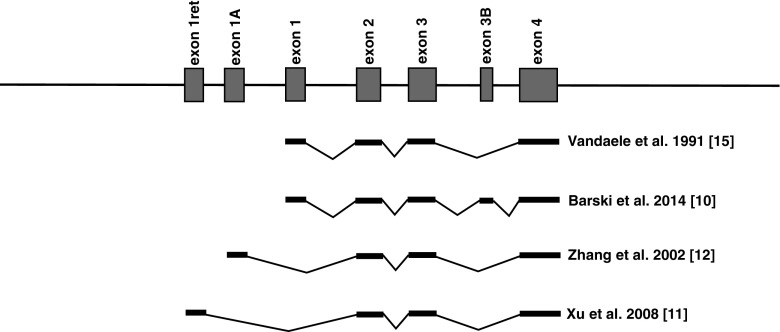
To date identified splice variants of the pcp2 gene

The gene coding pcp2 is located on the mouse chromosome 8 [4, 5]. Initial studies of the gene structure described four exons [6, 7]. These exons cover approximately 6 % of the gene locus spanning 8 kb. Transcription results in mRNA encoding a protein of approximately 16 kDa [2]. Translation start is at the ATG codon in exon 2 and the reading frame includes the entire exon 3 and part of exon 4. Mature pcp2 proteins incorporate a G protein-regulatory motif (GPR), the GoLoco motif [8, 9]. Experiments on the biochemical properties of pcp2 revealed its modulatory role in GDP release from G_i_ and G_o_, and the physical interaction with G subunits [8, 10]. Later experiments confirmed the existence of three further exons present when the gene structure is alternatively spliced [9, 10]. Alternatively spliced pcp2 variants give rise to three proteins of different molecular weight with different biochemical properties and possible differences in function [8, 11, 12] (Fig. 1).

Importantly, pcp2 is characterized by its very specific expression exclusively in PCs and retinal bipolar neurons [13, 14]. Expression of pcp2 starts within the first postnatal week and can be easily detected in Purkinje cells with Northern blotting [2, 3], in situ hybridization [2, 3, 10], Western blotting [13, 14], and immunohistochemistry [13, 14].

After discovery of the pcp2 gene a long time has passed until the final construct for Purkinje cell specific recombination has been designed. The overview of experiments and steps which resulted in this construct was presented in an earlier paper [1]. In the first instance, regulatory sequences of the pcp2 gene were used for expression of heterologous genes in Purkinje cell populations [1].

In the following text, we will focus on the use of the pcp2 sequence for targeting of PCs since the last summary published in 2002 [1].

## Purkinje Cell Specific Gene Targeting

The possibility of cell or tissue specific deletion of a gene has two big advantages:

- The ability to study consequences of knockout in a defined cell population. General knockouts almost always bring a question about the origin of an observed phenotype.
- The ability to study effects of knockouts that are lethal if switched off in the entire organism.

**Fig. 2:**
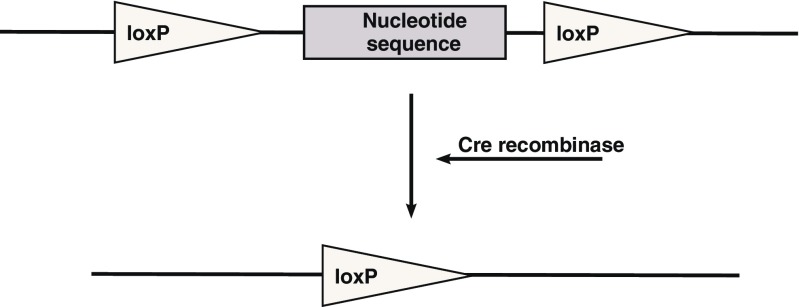
Diagram showing the principle of Cre/loxP genomic sequence excision

**Fig. 3:**
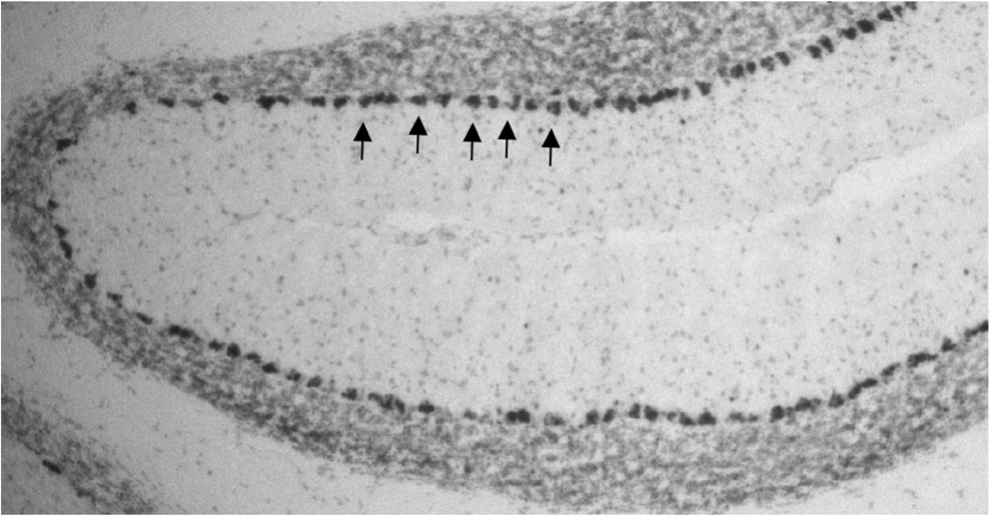
Expression of β-galactosidase in Purkinje cells (*black arrows*) after crossing of B6.129-Tg(Pcp2-cre)2Mpin/J with the reporter strain—B6.129S4-*Gt(ROSA)26Sortm1Sor/J*

The mostly used system for tissue or cell specific deletion of genes is the Cre/loxP system discovered in 1981 by Sternberg and Hamilton [15]. The system requires two components: a DNA sequence marked with the loxP sites—“floxed”, and expression of the Cre recombinase that recognizes the loxP sites and recombines (removes) the sequence between them. (Fig. 2). If the expression of recombinase is targeted onto a specific cell population, the deletion occurs only in these cells. To prove the specificity of the expression, a so called reporter mouse strain can be utilized. In these transgenic animals, a sequence coding for an easily detectable marker (chromogenic or fluorescent) is inserted into a locus characterized by ubiquitous expression. However, before the translation start, there is a STOP cassette floxed with loxP sites arresting the translation. If we then cross the reporter mouse with a mouse housing a cell-specific Cre expression, we are able to observe the chromogen or the fluorescence in the cells where recombination occurs (Fig. 3). Another recombination system accessible for gene targeting is based on the Flp recombinase [16]. This system can be combined with the Cre/loxP system to achieve recombination restricted to a subpopulation of cells. Both systems can be additionally modified by use of specifically designed inducible promoters. For more details see [1]

**Fig. 4:**
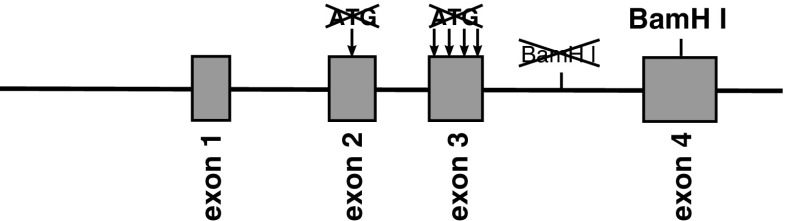
Diagram of the L7ΔAUG minigene. Note all mutated ATG and BamHI site. For easy insertion of nucleotide sequences there is the BamHI site in exon 4

A big step forward in targeting of PCs was the generation of the L7ΔAUG minigene by the John Oberdick group [17]. This construct was designed for easy insertion of DNA or cDNA sequences to express them specifically in Purkinje cells (Fig. 4). Because all transcription starts were removed from the pcp2 coding sequences, it became possible to express the protein of choice only with the use of a co-inserted ATG, using only regulatory capacities of the construct. Accessibility of this very convenient tool enabled us to generate a transgenic mouse strain with Purkinje cell specific expression of Cre recombinase in 2000 [18]. In 2004 and 2005, the generation of another two transgenic strains were reported, which expressed Cre recombinase using regulatory elements of the pcp2 gene. The first of them was generated by means of an artificial BAC chromosome [19], and the other one using the knock-in approach with the help of homologous recombination [20]. The primary strain published in 2000 [18] is accessible at The Jackson Laboratory under the strain name: B6.129-Tg(Pcp2-cre)2Mpin/J. The BAC-based strain can be provided also by JAX under the name B6.Cg-Tg(Pcp2-cre)3555Jdhu/J. The third one, the knock-in strain seems to be available only directly from the lab of origin. The first created strain [18] became the most popular and since its establishment was applied over 60 times for genetic manipulations in murine cerebellum.

The use of pcp2Cre based genetic manipulations started in 2003. For the first time the pcp2Cre strain [18] was used by the M. Meyer’s group for Purkinje cells specific knockout of calbindin D-28 k [21, 22]. The strain was created for this particular experiment to confirm the role of calbindin D-28 k in physiology of these neurons. Lack of calbindin D-28 k resulted in a very specific phenotype with clear signs of ataxia, however without signs of LTD disturbances. Detailed analysis of mutants revealed alteration in the early synaptic calcium transients (ESCT) without affecting the delayed synaptic calcium transients (DSCT). It is broadly accepted the DSCT are responsible for LTD, which seems to be independent from the calbindin D-28 k expression. Presence of LTD was additionally confirmed by means of behavioral tests verifying motor-learning.

In the following text, we will briefly review all applications of all three pcp2Cre strains (Table 1).

**Table 1:** Summary of studies utilizing the pcp2 for Cre based Purkinje cell specific transgenesis

	Publication details	Used Cre strain	Recombination effect	Experimental results
1.	Barski JJ, Hartmann J, et al. (2003) Calbindin in cerebellar Purkinje cells is a critical determinant of the precision of motor coordination. J Neurosci 23:3469-3477	B6.129-Tg(Pcp2-Cre)2Mpin/J [19]	Calbindin-D28k knockout	Changes in Ca2+ signaling resulting in altered early synaptic calcium transients (ECST), impaired motor-coordination, changes in visual reflexes without changes in LTD.
2.	Feil R, Hartmann J, et al. (2003) Impairment of LTD and cerebellar learning by Purkinje cell-specific ablation of cGMP-dependent protein kinase I. J Cell Biol 163:295-302	B6.129-Tg(Pcp2-Cre)2Mpin/J [19]	cGMPdependent protein kinase type I knockout	Strongly reduced LTD and impaired adaptation of the vestibulo-ocular reflex (VOR).
3.	Schaefer A, O’Carroll D, et al. (2007) Cerebellar neurodegeneration in the absence of microRNAs. Journal Exp Med 204:1553-1558	B6.Cg-Tg(Pcp2-cre)3555Jdhu/J [20]	Dicer knockout	Progressive loss of miRNAs, followed by cerebellar degeneration and development of ataxia.
4.	Adelman C.A., De S., et al. (2009) Rad50 is dispensable for the maintenance and viability of postmitotic tissues. Molecular and Cellular Biology, vol. 29, no. 2, p.483-492	B6.129-Tg(Pcp2-Cre)2Mpin/J [19]	Rad50 knockout	No alteration in cerebellar size or architecture, no signs of akinesisor balance abnormalities, only minor gait abnormalities were observed.
5.	Kim JC, Cook MN, et al. (2009) Linking Genetically Defined Neurons to Behavior through a Broadly Applicable Silencing Allele. Neuron 63:305-315	B6.129-Tg(Pcp2-Cre)2Mpin/J [19]	Delivery of tetanus toxin to inhibit the vesicular neurotransmission	Cell type specific inhibition of exocytosis at the granule cell/Purkinje cell synapses, followed by motor-coordination impairment.
6.	Lorenzetto E, Caselli L, et al. (2009) Genetic perturbation of postsynaptic activity regulates synapse elimination in developing cerebellum. PNAS 106:16475-16480	B6.129-Tg(Pcp2-Cre)2Mpin/J [19]	Conditional overexpression of a muscle chloride channel-YFPfusion protein (Thy1-SCLY	Decreased excitability of postnatal Purkinje cells resulting in persistent climbing fiber innervation of Purkinje cells and disturbed redistribution of climbing fiber/Purkinje cells synapses.
7.	Wulff P, Schonewille M, et al. (2009) Synaptic inhibition of Purkinje cells mediates consolidation of vestibulo-cerebellar motor learning. Nat Neurosci 12:1042-U118	B6.129-Tg(Pcp2-Cre)2Mpin/J [19]	Ablation of the γ2 subunit, of the GABA_A_ receptor	Loss of spontaneous inhibitory postsynaptic currents (sIPSCs), changes in temporal pattern of Purkinje cell simple spikes, partial impairment of motor performance as stated by vestibulo-ocular reflex (VOR) and optokinetic reflex (OKR) analysis.
8.	Belmeguenai A, Hosy E, et al. (2010) Intrinsic Plasticity Complements Long-Term Potentiation in Parallel Fiber Input Gain Control in Cerebellar Purkinje Cells. J Neurosci 30:13630-13643	B6.129-Tg(Pcp2-Cre)2Mpin/J [19]	Calcineurin knockout	Calcineurin deficiency resulting in impaired LTP and intrinsic plasticity of Purkinje cells without affecting of LTD
9.	Briatore F, Patrizi A, et al. (2010) Quantitative Organization of GABAergic Synapses in the Molecular Layer of the Mouse Cerebellar Cortex. PLoS ONE 5	B6.129-Tg(Pcp2-Cre)2Mpin/J [19]	Ablation of the α1 subunit of the GABA_A_ receptor	Complete loss of the GABA_A_ receptors in Purkinje cells.
10.	Chen X, Kovalchuk Y, et al. (2010) Disruption of the olivo-cerebellar circuit by Purkinje neuron-specific ablation of BK channels. Proceedings of the PNAS 107:12323-12328	B6.129-Tg(Pcp2-Cre)2Mpin/J [19]	Ablation of the large-conductance voltage- and Ca2^+^-activated K^+^ channels (BK channels)	Decrease of single and complex spike activity, disruption of the olivo-cerebellar circuit, ataxic gait, and impaired motor-coordination.
11.	Elrick MJ, Pacheco CD, et al. (2010) Conditional Niemann-Pick C mice demonstrate cell autonomous Purkinje cell neurodegeneration. Hum Mol Genet 19:837-847	B6.129-Tg(Pcp2-Cre)2Mpin/J [19]	Deletion of the *Npc1*	Degeneration of Purkinje cells followed by tremor and motor impairment assessed by rotarod and balance beam.
12.	Satz JS, Ostendorf AP, et al. (2010) Distinct Functions of Glial and Neuronal Dystroglycan in the Developing and Adult Mouse Brain. J Neurosci 30:14560-14572	B6.129-Tg(Pcp2-Cre)2Mpin/J [19]	Dystroglycan knockout	Ablation of the α-dystroglycan in Purkinje cells.
13.	Schonewille M, Belmeguenai A, et al. (2010) Purkinje Cell-Specific Knockout of the Protein Phosphatase PP2B Impairs Potentiation and Cerebellar Motor Learning. Neuron 67:618-628	B6.129-Tg(Pcp2-Cre)2Mpin/J [19]	Protein phosphatase PP2B knockout	Loss of LTP in Purkinje cells, impaired vestibulo-ocular reflex (VOR) and eyeblink conditioning.
14.	Smith-Hicks C, Xiao B, et al. (2010) SRF binding to SRE 6.9 in the Arc promoter is essential for LTD in cultured Purkinje cells. Nature Neurosci 13:1082-1U73	B6.129-Tg(Pcp2-Cre)2Mpin/J [19]	Serum responding factor (SRF) knockout	Lack of the late phase of parallel-Purkinje cells LTD.
15.	Wall NR, Wickersham IR, et al. (2010) Monosynaptic circuit tracing in vivo through Cre-dependent targeting and complementation of modified rabies virus. PNAS 107:21848-21853	B6.129-Tg(Pcp2-Cre)2Mpin/J [19]	Cre dependent expression of viruses	Establishment of a method for retrograde tracing of presynaptic cells
16.	Wang M, Ye R, et al. (2010) Essential role of the unfolded protein response regulator GRP78/BiP in protection from neuronal apoptosis. Cell Death Differ 17:488-498	B6.129-Tg(Pcp2-Cre)2Mpin/J [19]	GRP78/BiP knockout	Retarded growth of animals, severe motor coordination impairment and cerebellar atrophy
17.	Furrer SA, Mohanachandran MS, et al. (2011) Spinocerebellar Ataxia Type 7 Cerebellar Disease Requires the Coordinated Action of Mutant Ataxin-7 in Neurons and Glia, and Displays Non-Cell-Autonomous Bergmann Glia Degeneration. J Neurosci 31:16269-16278	B6.129-Tg(Pcp2-Cre)2Mpin/J [19]	Inactivation of polyQ-ataxin-7 expression	Partial improvement of SCA7 phenotype
18.	Gutierrez DV, Mark MD, et al. (2011) Optogenetic Control of Motor Coordination by G(i/o) Protein-coupled Vertebrate Rhodopsin in Cerebellar Purkinje Cells. J Biol Chem 286:25848-25858	B6.129-Tg(Pcp2-Cre)2Mpin/J [19]	Optogenetic modulation of the Gi/o activity in Purkinje cells	Reduction of simple spikes with concomitant changes in motor behavior.
19.	Jaarsma D, van der Pluijm I, et al. (2011) Age-Related Neuronal Degeneration: Complementary Roles of Nucleotide Excision Repair and Transcription-Coupled Repair in Preventing Neuropathology. Plos Genetics 7	B6.129-Tg(Pcp2-Cre)2Mpin/J [19]	Purkinje cell-specific Xpa gene inactivation in Csb null-mutants	Purkinje cell loss and other degeneration signs: argyrophilic axonal degeneration in the areas that contain PCs, argyrophilic debris in molecular and PCs layers with a strong increasement in GFAP-immunoreactivity. Strong nuclear ATF3 staining in about 1 % of Purkinje cells and infrequent caspase-3 positive cells.
20.	Kalinovsky A, Boukhtouche F, et al. (2011) Development of axon-target specificity of ponto-cerebellar afferents. PLoS Biol 9:e1001013	L7-Cre knock-in mouse [21]	Purkinje cell-specific ablation of bone morphogenetic protein 4 (BMP4)	Exuberant mossy fiber-Purkinje cell interactions.
21.	Kumar R, Hunt CR, et al. (2011) Purkinje cell-specific males absent on the first (mMof) gene deletion results in an ataxia-telangiectasia-like neurological phenotype and backward walking in mice. Proceedings of the National Academy of Sciences of the United States of America 108:3636-3641	B6.129-Tg(Pcp2-Cre)2Mpin/J [19]	Purkinje cell-specific deletion of the mouse males absent on the first (mMof) gene	Impaired motor coordination, ataxia, a backward-walking phenotype, and a reduced life span
22.	Lo L, Anderson DJ (2011) A Cre-Dependent, Anterograde Transsynaptic Viral Tracer for Mapping Output Pathways of Genetically Marked Neurons. Neuron 72:938-950	B6.129-Tg(Pcp2-Cre)2Mpin/J [19]	Cre dependent expression of viruses	Establishment of a method for anterograde trans-synaptic tracing for mapping of synaptic outputs.
23.	Mark MD, Maejima T, et al. (2011) Delayed Postnatal Loss of P/Q-Type Calcium Channels Recapitulates the Absence Epilepsy, Dyskinesia, and Ataxia Phenotypes of Genomic Cacna1A Mutations. J Neurosci 31:4311-4326	B6.129-Tg(Pcp2-Cre)2Mpin/J [19]	Delayed deletion of the P/Q-type channel in Purkinje cells	Loss of the channel in PCs at sixth day of age cause ataxia, episodic dyskinesia and absence epilepsy. Delayed postnatal loss of the P/Q-type channel is sufficient to trigger full disease phenotype.
24.	Reith R, Way S, et al. (2011) Loss of the tuberous sclerosis complex protein tuberin causes Purkinje cell degeneration. Neurobiology of Disease 43:113-122	B6.129-Tg(Pcp2-Cre)2Mpin/J [19]	Tsc2-null Purkinje cells	The loss of Tsc2 lead to progressive increase in Purkinje cell size, their death, and apoptosis.
25.	Xu P, Das M, et al. (2011) JNK regulates FoxO-dependent autophagy in neurons. Genes & Development 25:310-322	B6.129-Tg(Pcp2-Cre)2Mpin/J [19]	Triple neuronal deficiency of JNK1, JNK2, JNK3	Purkinje cells exhibit reduced dendritic arborization but can function without the JNK signaling pathway.
26.	Yu T, Shakkottai VG, et al. (2011) Temporal and cell-specific deletion establishes that neuronal Npc1 deficiency is sufficient to mediate neurodegeneration. Hum Mol Genet 20:4440-4451	B6.129-Tg(Pcp2-Cre)2Mpin/J [19]	Purkinje cell-specific *Npc1* null mutant	Deletion of Npc1 in neurons is sufficient to cause neurological symptoms in Niemann-Pick type C disease.
27.	Zhang L, Yokoi F, et al. (2011) Altered Dendritic Morphology of Purkinje cells in Dyt1 Delta GAG Knock-In and Purkinje Cell-Specific Dyt1 Conditional Knockout Mice. PLoS ONE 6:	B6.129-Tg(Pcp2-Cre)2Mpin/J [19]	*Dyt1* knockout	Shortened primary large dendrites and decreased spines on the distal dendrites of Purkinje cells.
28.	Almajan ER, Richter R, et al. (2012) AFG3L2 supports mitochondrial protein synthesis and Purkinje cell survival. Journal of Clinical Investigation 122:4048-4058	B6.129-Tg(Pcp2-Cre)2Mpin/J [19]	*Afg3l2* knockout	Purkinje cells show respiratory dysfunctions, decreased rate of mitochondrial protein synthesis associated with impaired mitochondrial ribosome assembly, neurodegeneration, and secondary inflammation.
29.	Buttermore ED, Piochon C, et al. (2012) Pinceau Organization in the Cerebellum Requires Distinct Functions of Neurofascin in Purkinje and Basket Neurons during Postnatal Development. J Neurosci 32:4724-4742	B6.129-Tg(Pcp2-Cre)2Mpin/J [19]	*Nfasc* knockout	Disturbed maturation of the Purkinje cell’s axon initial site, loss of Purkinje neuron spontaneous activity and pinceau disorganization, Purkinje cell degeneration, and ataxia.
30.	Duran J, Florencia Tevy M, et al. (2012) Deleterious effects of neuronal accumulation of glycogen in flies and mice. Embo Molecular Medicine 4:719-729	B6.129-Tg(Pcp2-Cre)2Mpin/J [19]	Cre dependent expression of *mMGS-9A*	Glycogen accumulation, loss of Purkinje cells and ataxia.
31.	Gehman LT, Meera P, et al. (2012) The splicing regulator Rbfox2 is required for both cerebellar development and mature motor function. Genes & Development 26:445-460	B6.129-Tg(Pcp2-Cre)2Mpin/J [19]	*Rbfox1* and *Rbfox2* knockout	Highly irregular firing of Purkinje cells caused by improperly spliced the Scn8a mRNA which encodes the Nav1.6 sodium channel.
32.	Kageyama Y, Zhang Z, et al. (2012) Mitochondrial division ensures the survival of postmitotic neurons by suppressing oxidative damage. J Cell Biol 197:535-551	B6.129-Tg(Pcp2-Cre)2Mpin/J [19]	*Drp1* knockout	Neurodegeneration caused by mitochondrial alterations: excess fusion, oxidative damage, accumulation of ubiquitin and mitophagy markers, and loss of respiratory function.
33.	Kullmann J, Neumeyer A, et al. (2012) Purkinje Cell Loss and Motor Coordination Defects in Profilin1 Mutant Mice. Neuroscience 223:355-364	B6.129-Tg(Pcp2-Cre)2Mpin/J [19]	*Profilin-1* knockout	Unchanged cytoarchitecture of the Purkinje cell layer
34.	Lefebvre JL, Kostadinov D, et al. (2012) Protocadherins mediate dendritic self-avoidance in the mammalian nervous system. Nature 488:517-521	B6.Cg-Tg(Pcp2-cre)3555Jdhu/J [20]	*Pcdhgs* knockout	Disorganized arbors and often each other crossed dendrites with no detectable effect on survival, shape, size or branching pattern of Purkinje cells.
35.	Lu S, Lu LY, et al. (2012) Cerebellar defects in Pdss2 conditional knockout mice during embryonic development and in adulthood. Neurobiology of Disease 45:219-233	B6.Cg-Tg(Pcp2-cre)3555Jdhu/J [20]	*Pdss2* knockout	Loss of Purkinje cells and ataxia
36.	Peng C, Yan S, et al. (2012) Vps18 deficiency inhibits dendritogenesis in Purkinje cells by blocking the lysosomal degradation of Lysyl Oxidase. Biochem Biophys Res Commun 423:715-720	B6.129-Tg(Pcp2-Cre)2Mpin/J [19]	*Vps18* knockout	Impairment of the balance and coordination ability. Loss of Purkinje cells and unchanged dendrites.
37.	Peng C, Ye J, et al. (2012) Ablation of Vacuole Protein Sorting 18 (Vps18) Gene Leads to Neurodegeneration and Impaired Neuronal Migration by Disrupting Multiple Vesicle Transport Pathways to Lysosomes. Journal of Biological Chemistry 287:32861-32873	B6.129-Tg(Pcp2-Cre)2Mpin/J [19]	*Vps18* knockout	Loss of Purkinje cells
38.	Seja P, Schonewille M, et al. (2012) Raising cytosolic Cl- in cerebellar granule cells affects their excitability and vestibulo-ocular learning. EMBO J 31:1217-1230	B6.129-Tg(Pcp2-Cre)2Mpin/J [19]	*Kcc2* knockout	Decrease in the gain of the optokinetic reflex, impairment in vestibule-ocular reflex decrease learning and deficits in gain consolidation.
39.	Todorov B, Kros L, et al. (2012) Purkinje Cell-Specific Ablation of Ca(V)2.1 Channels is Sufficient to Cause Cerebellar Ataxia in Mice. Cerebellum 11:246-258	B6.129-Tg(Pcp2-Cre)2Mpin/J [19]	CaV2.1-α1A-knockout	Ataxia and Purkinje cell loss.
40.	Tsai PT, Hull C, et al. (2012) Autistic-like behaviour and cerebellar dysfunction in Purkinje cell Tsc1 mutant mice. Nature 488:647-+	B6.129-Tg(Pcp2-Cre)2Mpin/J [19]	Tsc1 knockout	Autistic-like behaviors, including abnormal social interaction, repetitive behavior, and vocalizations, in addition to decreased PC excitability
41.	Yokoi F, Mai TD, et al. (2012) Improved motor performance in Dyt1 Delta GAG heterozygous knock-in mice by cerebellar Purkinje-cell specific Dyt1 conditional knocking-out. Behavioural Brain Research 230:389-398	B6.129-Tg(Pcp2-Cre)2Mpin/J [19]	Purkinje cell specific *Dyt1* knockout	Despite normal gait, ultrastructure of nuclear envelopes in Purkinje cells, and synapse formation in molecular layers, mutant mice exhibit enhanced motor coordination in the beam-walking test.
42.	Yokoi F, Dang MT, et al. (2012) Abnormal nuclear envelope in the cerebellar Purkinje cells and impaired motor learning in DYT11 myoclonus-dystonia mouse models. Behavioural Brain Research 227:12-20	B6.129-Tg(Pcp2-Cre)2Mpin/J [19]	Paternally inherited cerebellar Purkinje cell-specific *Sgce* conditional knockout	Motor learning deficits, without motor deficits and myoclonus Nuclear envelope in the cerebellar Purkinje cells without abnormalities.
43.	Zariwala HA, Borghuis BG, et al. (2012) A Cre-Dependent GCaMP3 Reporter Mouse for Neuronal Imaging In Vivo. J Neurosci 32:3131-3141	B6.129-Tg(Pcp2-Cre)2Mpin/J [19]	GCaMP3 reporter mouse (Ai38)	Cre dependent GCaMP3 expression in Purkinje cells. Method for neuronal imaging.
44.	Asrican B, Augustine GJ, et al. (2013) Next-generation transgenic mice for optogenetic analysis of neural circuits. Frontiers in Neural Circuits 7	B6.129-Tg(Pcp2-Cre)2Mpin/J [19]	Mouse line PCP2-Cre-ChR2	ChR2 expression in Purkinje cells. An optogenetic method.
45.	Chaumont J, Guyon N, et al. (2013) Clusters of cerebellar Purkinje cells control their afferent climbing fiber discharge. PNAS 110:16223-16228	B6.129-Tg(Pcp2-Cre)2Mpin/J [19]	Cre dependent expression of ChR2-eYFP	Expression of the ChR2-YFP fusion protein in Purkinje cells. Transgene expression does not perturb motor functions.
46.	de Graaf EL, Vermeij WP, et al. (2013) Spatio-temporal Analysis of Molecular Determinants of Neuronal Degeneration in the Aging Mouse Cerebellum. Molecular & Cellular Proteomics 12:1350-1362	B6.129-Tg(Pcp2-Cre)2Mpin/J [19]	*Ercc1* knockout	Neuron morphological changes, neuron degeneration, inflammation, and behavior disorders (motor function decline, lack of capacity in motoric learning).
47.	Ju X, Wen Y, et al. (2013) The role of p38 in mitochondrial respiration in male and female mice. Neurosci Lett 544:152-156	B6.129-Tg(Pcp2-Cre)2Mpin/J [19]	p38 knockout	Suppression of the mitochondrial respiration in males. Increase in COX expression in female.
48.	Reith R, McKenna J, et al. (2013) Loss of Tsc2 in Purkinje cells is associated with autistic-like behavior in a mouse model of tuberous sclerosis complex. Neurobiology of Disease 51:93-103	B6.129-Tg(Pcp2-Cre)2Mpin/J [19]	Tsc2 knockout	Loss of Purkinje cells, autistic-like behavior.
49.	Rodrigues PM, Grigaravicius P, et al. (2013) Nbn and Atm Cooperate in a Tissue and Developmental Stage-Specific Manner to Prevent Double Strand Breaks and Apoptosis in Developing Brain and Eye. PLoS ONE 8.	B6.129-Tg(Pcp2-Cre)2Mpin/J [19]	*Nbn* and *Nbn/Atm* knockout	Nbn and Atm are dispensable for Purkinje cells homeostasis in mouse.
50.	Sugawara T, Hisatsune C, et al. (2013) Type 1 Inositol Trisphosphate Receptor Regulates Cerebellar Circuits by Maintaining the Spine Morphology of Purkinje Cells in Adult Mice. J Neurosci 33:12186-+	B6.129-Tg(Pcp2-Cre)2Mpin/J [19]	*IP3R1*knockout	Increase in spine density and spine length of Purkinje cells. Abnormal parallel fibers -Purkinje cells circuits and ataxia.
51.	Thomanetz V, Angliker N, et al. (2013) Ablation of the mTORC2 component rictor in brain or Purkinje cells affects size and neuron morphology. The Journal of Cell Biology 201:293-308	L7-Cre knock-in mouse [21]	*rictor* knockout	Increased cell body size. Diminished diameter of the primary dendrites and axons of Purkinje cells. Changes in synaptic functions.
52.	Achterberg KG, Buitendijk GH, et al. (2014) Temporal and Region-Specific Requirements of alpha CaMKII in Spatial and Contextual Learning. J Neurosci 34:11180-11187	B6.129-Tg(Pcp2-Cre)2Mpin/J [19]	*Camk2a* knockout	Cerebellar deletion of αCaMKII does not affect spatial learning and contextual fear learning.
53.	Alvarez-Saavedra M, De Repentigny Y, et al. (2014) Snf2h-mediated chromatin organization and histone H1 dynamics govern cerebellar morphogenesis and neural maturation. Nature Communications 5	B6.129-Tg(Pcp2-Cre)2Mpin/J [19]	*Snf2h* knockout	Snf2h ablation affects chromatin ultrastructure and dendritic arborisation but alters cognitive skills rather than motor control.
54.	Gibson DA, Tymanskyj S, et al. (2014) Dendrite Self-Avoidance Requires Cell-Autonomous Slit/Robo Signaling in Cerebellar Purkinje Cells. Neuron 81:1040-1056	B6.129-Tg(Pcp2-Cre)2Mpin/J [19]	*Robo2*knockout *Slit2* knockout	Dendrite self-avoidance alterations: perturbed spacing of neighboring branches and crossing defects. Gait alterations.
55.	Kayakabe M, Kakizaki T, et al. (2014) Motor dysfunction in cerebellar Purkinje cell-specific vesicular GABA transporter knockout mice. Frontiers in Cellular Neuroscience 7:	L7-Cre knock-in mouse [21]	*VGAT* knockout	Ataxia without Purkinje cell degeneration.
56.	Kruse W, Krause M, et al. (2014) Optogenetic Modulation and Multi-Electrode Analysis of Cerebellar Networks In Vivo. PLoS ONE 9:	B6.129-Tg(Pcp2-Cre)2Mpin/J [19]	Purkinje cell-specific expression of ChR2	PC firing precisely monitored and modulated by light-activation of channelrhodopsin-2 (ChR2) [optogenetic method]
57.	Rahmati N, Owens CB, et al. (2014) Cerebellar Potentiation and Learning a Whisker-Based Object Localization Task with a Time Response Window. J Neurosci 34:1949-1962	B6.129-Tg(Pcp2-Cre)2Mpin/J [19]	CNB1 knockout	Impairments in learning an object localization task and deficiencies in temporal tuning.
58.	Venkatraman A, Hu YS, et al. (2014) The histone deacetylase HDAC3 is essential for Purkinje cell function, potentially complicating the use of HDAC inhibitors in SCA1. Hum Mol Genet 23:3733-3745	B6.129-Tg(Pcp2-Cre)2Mpin/J [19]	Purkinje cell-specific HDAC3 null mutation	Purkinje cells degeneration, ataxia.
59.	Zhang L, Chung SK, et al. (2014) The knockout of secretin in cerebellar Purkinje cells impairs mouse motor coordination and motor learning. Neuropsychopharmacology 39:1460-1468	B6.Cg-Tg(Pcp2-cre)3555Jdhu/J [20]	SCT knockout	Impairments in neuromuscular strength, motor coordination, and motor learning abilities.
60.	Rose S.J., Kriener L.H., et al. (2014) The first knockin mouse model of episodic ataxia type 2. Experimental Neurology 261, 553-562	B6.129-Tg(Pcp2-Cre)2Mpin/J [19]	Purkinje cell-specific expression of a mutated form of CACNA1A gene	No overt ataxia signs, reduction in CaV2.1 current density in Purkinje cells.
61.	Szabo V, Ventalon C et al. (2014) Spatially selective holographic photoactivation and functional fluorescence imaging in freely behaving mice with a fiberscope. Neuron 17:1157-1169	B6.129-Tg(Pcp2-Cre)2Mpin/J [19]	Purkinje cell-specific activation of the Tg(CAG-Brainbow1.0)2Eggn transgene	Possibility of functional fluorescence imaging in spontaneously behaving mouse.
62.	Razavfsky D, Hodzic D (2015) A variant of Nesprin1 giant devoid of KASH domain underlies the molecular etiology of autosomal recessive cerebellar ataxia type I. Neurobiol Dis 78:57-67	B6.129-Tg(Pcp2-Cre)2Mpin/J [19]	Purkinje cell-specific expression of the Klarsicht/Anc1/Syne1 homology (KASH) domain	Inactivation of Nesprins without any cerebellar phenotype.
63.	Hoogland TM, De Gruijil JR et al. (2015) Role of Synchronous Activation of Cerebellar Purkinje Cell Ensembles in Multi-joint Movement Control. Curr Biol 25:115-1165	B6.129-Tg(Pcp2-Cre)2Mpin/J [19]	Purkinje cell-specific activation of the ChR2(H134R)-eYFP transgene	Optogenetic-based modulation of selected Purkinje cells resulting in changes of locomotion and postural behavior.

Experimental approaches and studies summarized in Table 1 show clearly, how useful is the possibility to specifically manipulate gene expression in the cerebellar PCs. Published data allow for the manipulation of PCs function in many aspects and extend our knowledge on cerebellar physiology. Most of the experiments are typical conditional knockouts where expression of a selected gene is switched off according to the spatial and temporal expression of the pcp2 gene. Some of the cited studies attempted to express conditionally selected genes using a similar approach as in the case of reporter mouse (Tab.1/5,6,15,18,22,30,43–45,56,60–63). In these experiments, 5′ to the coding sequence of nucleotides a floxed STOP cassette was placed. This kind of approach was also applied in the new field of conditional manipulation of neuron activity and namely in optogenetics (Tab.1/18, 44, 45, 56, 61, 63). This sophisticated method uses light to control activity of neurons or an individual neuron, which are sensitive light. The light sensitivity results from transgenic manipulations targeting expression of light-sensitive proteins to neurons. Optogenetics enables researchers to influence the activity of neurons in living animals, allowing for the precise recording of effects in real time.

Another group of experiments aimed to express heterological genes in PCs through the application of regulatory elements of pcp2 (Table 2).

**Table 2:** Summary of studies utilizing the pcp2 regulatory elements for Purkinje cell specific expression of heterologous genes

	Publication details	Experimental approach	Mutation effect
1.	Zhang X, Baader SL, et al. (2001) High level Purkinje cell specific expression of green fluorescent protein in transgenic mice. Histochem Cell Biol 115:455-64	Purkinje-cell specific of GFP	Homogenous and stable expression of GFP in cerebellar Purkinje cells.
2.	Zu T, Duvick LA, et al. (2004) Recovery from Polyglutamine-Induced Neurodegeneration in Conditional SCA1 Transgenic Mice. J Neurosci 24:8853-8861	Purkinje-cell specific tetracyclin transactivator expression	Purkinje-cell specific inactivation of the ataxin transgene. Partial reversal of ataxia type 1 manifestations.
3.	Wang T, Parris J, et al. (2006) The carboxypeptidase-like substrate-binding site in Nna1 is essential for the rescue of the Purkinje cell degeneration (pcd) phenotype. Mol Cell Neurosci 33:200-213	Purkinje-cell specific expression of Nna1, a protein with metallocarboxypeptidase domain	Partial rescue of ataxia and Purkinje cells degeneration in the *pcd* strain mice.
4.	Echigo R, Nakao K, et al. (2009) Generation of L7-tTA knock-in mice. Kobe J Med Sci 54:E272-E278	Purkinje-cell specific tetracyclin transactivator expression via a knock-in approach	Stable and cell specific expression of tTA under the pcp2 regulatory elements
5.	Chang YC, Lin CY, et al. (2011) Neuroprotective effects of granulocyte-colony stimulating factor in a novel transgenic mouse model of SCA17. J Neurochem 118:288-303	Purkinje-cell specific expression of mutated form of the *TATA-binding protein (hTBP)* gene	Generation of an animal model of spinocerebellar ataxia type 17 (SCA17).
6.	Wagner W, McCroskery S, et al. (2011) An efficient method for the long-term and specific expression of exogenous cDNAs in cultured Purkinje neurons. J Neurosci Meth 200:95-105	Generation of plasmid transfection system suitable for cell specific expression of various cDNAs in cultured Purkinje cells	Stable and long-term expression of series of fluorescent proteins in Purkinje cells.
7.	Fujita-Jimbo E, Momoi T (2014) Specific expression of FOXP2 in cerebellum improves ultrasonic vocalization in heterozygous but not in homozygous Foxp2 (R552H) knock-in pups. Neurosci Lett 566:162-166	Purkinje-cell specific expression of humanFOXP2-myc in mouse cerebellum	Partial rescue of proper ultrasonic vocalizations in FOXP mutant mouse, animal model of language disorders.

Among these experiments, we can find four, which have important methodological input in the field of cerebellum research (Tab.2/1,2,4,6). Two of them resulted in stable expression of fluorescent proteins in PCs – GFP, Cerulean and Cherry (Tab.2/1,6). Another two experiments enabled researchers to specifically express a tetracyclin transactivator in these neurons (Tab.2/2,5).

In the following, we will discuss shortly results of selected papers, which are the most interesting from the authors point of view.

Shortly, after M. Meyer’s group report [18] another work concerning motor learning using conditional knockout was published by Feil et al. in 2003. Investigators created a mouse without cGMP-dependent kinase I in PCs to study its role in LTD and cerebellar learning. Researchers emphasized, that cell specific ablation not only gave opportunity to overcome unwanted effect of conventional gene targeting which affects whole organism but also was privileged in that study because pharmaceutical tools had limitations. Firstly, there were no acknowledged specific cGKI inhibitor and secondly there was concern about the exaggeration of the importance of some pathways that might be less important, or even not used under physiological conditions. Tests revealed strongly reduced LTD and impaired adaptation of the vestibulo-ocular reflex in mutants. Results also showed a specific, indispensable role of cGPKI signaling in cerebellar LTD and motor learning. CGKI seems also necessary for motor coordination. Study strongly supports the conception that cerebellar LTD is involved in a specific form of motor learning but not in motor performance in general [23].

A group of publications investigating developmental relationships and refinement of neuronal connections have been initiated by Lorenzetto et al. In 2009, researchers employed a conditional overexpression of muscle chloride channel-YFP fusion protein by means of Cre-loxP system exclusively in PCs and provide a new tool to verify the role electrical activity for elimination of supernumerary climbing fiber inputs. Their new approach had an advantage over previous methods—it allows to verify a single cell type specific perturbation instead of rather global effects of a particular neurotransmitter or signaling system malfunction. The study brought a strong evidence that synapse elimination in the CNS needs appropriate electrical activity in the postsynaptic cells [24].

One of the most sophisticated cre/loxP-based methods has been developed and described by Wall et al. In 2010, they published a work about the new tool for retrograde tracing of presynaptic cells. They used two viruses—one the avian sarcoma leucosis virus glycoprotein EnvA—pseudotyped rabbies virus that was capable to infect only cells expressing an avian receptor protein TVA. The second one was the cre-dependent helper virus that contained genes of the abovementioned TVA and the rabies glycoprotein B19G. This allowed the first one virus to spread retrogradely. Moreover, the EnvA-pseudotyped rabbies virus had no B19G gene so was unable to spread in the absence of another source of that glycoprotein. Combination of these viruses with the concomitant cell specific cre expression gave an ability to identify the direct local and long-distance connections to Cre-expressing neurons in vivo [25].

Another group of studies focused on PCs employed an optogenetic approach. First of the papers we would like to focus on is from 2011. Gutierrez et al. created an optogenetic mouse model for the cell specific expression of vertebrate rhodopsin (vRh) to investigate the functional impact of Gi/o protein mediated modulation on the cerebellar function via the spike modulation in PCs. First, they created mice with a cell-specific expression of vRh-GFP. Secondly, investigators showed that light activation of vRh selectively expressed in PCs, similarly to GABAbR activation by baclofen in PCs, reduces the firing frequency of PCs in vivo. At the end, they conducted series of motor tests confirming that Gi/o-mediated modulation of PC firing alters motor coordination in behaving mice [26].

Comprehensive study of Asrican et al. describes new mouse lines useful for investigation of neural circuits. Researchers compared three strategies for expressing channelrhodopsin-2 (ChR2) in PCs. Pcp-2 promoter was used to target the expression of ChR2 with a H134 mutation and the method was the most selective to Purkinje neurons. The other two lines utilized the parvalbumin promoter and targeted ChR2 expression additionally to other neurons of the cerebellar cortex. Histological analysis revealed significant expression of targeted protein in the cerebellum of all three lines. In contrast to pcp-2-based line where ChR2 expression was present only in Purkinje neurons, other two lines displayed expression of ChR2 also in molecular layer interneurons. The study demonstrated the convergence of a variable number of Purkinje neurons input onto the deep cerebellar nuclei neurons. Authors emphasized that pcp2-based line was ideally suited for PC circuits mapping by means of the optogenetic method [27].

Other application of Purkinje cell specific ChR2 expression was showed by Kruse et al. in 2014. To analyze neuronal networks Kruse with colleagues employed similar method to precisely monitor and modulate PCs firing but their method was improved by a more targeted application of light. Researchers found that PCs switched on and off in a milliseconds time resolution while being light activated. Same effect on PCs was brought by photo stimulation of molecular layer interneurons, whereas granule cells activation only slowly modulated its firing. These results indicate on the functional modulation of PCs firing rate by accompanying cells [28].

Interesting research opportunities brought also the work of Szabo et al. Their method allowed to control and monitor dynamics of microcircuits in freely behaving mice with almost cellular level resolution [29].

Last one of the most interesting optogenetic experiments in this review comes from Hoogland et al. from May 2015. In this study, mice with mutated P/Q-type calcium channels were subjected to the motor coordination test on a disk treadmill to study motor behavior and then to find correlations between the activity of PC ensembles and motor performance parameters. Optogenetics was applied to evoke simple spike activity simultaneously in multiple Purkinje neurons expressing ChR2(H134R)-eYFP driven by pcp2-Cre. Combination of these two methods enabled to characterize walking patterns, and preferred sequences of limb placement after light-induced simple spike activity of PCs groups at rest. During locomotion various gait-inhibition patterns caused by PCs activation could be revealed. Study showed that optogenetically evoked simple spike activity in PC groups can influence the sequence and timing of limb and body movements at rest and during locomotion; output of cerebellar zones in contribution with other downstream regions is involved into coordination of multi-joint and multi-limb-mediated movements [30].

Since the publication of the first two papers describing pcp2, there has been a substantial increase in the understanding of cerebellar physiology [2, 3]. It became easier to study cerebellar function by selectively manipulating its sole output—Purkinje cells. The big step forward however was the possibility to switch off genes with putative importance PC physiology and possibility to manipulate activity of their function without affecting neighboring neuronal elements. Purkinje cell specific gene manipulation makes it possible to analyze even single Purkinje cells with the use one of the very modern techniques—optogenetics.
